# Direct Growth of Bismuth Film as Anode for Aqueous Rechargeable Batteries in LiOH, NaOH and KOH Electrolytes

**DOI:** 10.3390/nano5041756

**Published:** 2015-10-22

**Authors:** Wenhua Zuo, Pan Xu, Yuanyuan Li, Jinping Liu

**Affiliations:** 1 School of Chemistry, Chemical Engineering and Life Science and State Key Laboratory of Advanced Technology for Materials Synthesis and Processing, Wuhan University of Technology, Wuhan 430070, China; E-Mails: zuowh1991@outlook.com (W.Z.); xupan2013@126.com (P.X.); 2 Institute of Nanoscience and Nanotechnology, Department of Physics, Central China Normal University, Wuhan 430079, China; 3 School of Optical and Electronic Information, Huazhong University of Science and Technology, Wuhan 430074, China

**Keywords:** aqueous rechargeable batteries, Bi, anode, EIS, ion diffusion coefficient

## Abstract

As promising candidates for next-generation energy storage devices, aqueous rechargeable batteries are safer and cheaper than organic Li ion batteries. But due to the narrow voltage window of aqueous electrolytes, proper anode materials with low redox potential and high capacity are quite rare. In this work, bismuth electrode film was directly grown by a facile hydrothermal route and tested in LiOH, NaOH and KOH electrolytes. With low redox potential (reduction/oxidation potentials at *ca.* −0.85/−0.52 V *vs.* SCE, respectively) and high specific capacity (170 mAh·g^−1^ at current density of 0.5 A·g^−1^ in KOH electrolyte), Bi was demonstrated as a suitable anode material for aqueous batteries. Furthermore, by electrochemical impedance spectroscopy (EIS) analysis, we found that with smaller *R*_s_ and faster ion diffusion coefficient, Bi electrode film in KOH electrolyte exhibited better electrochemical performance than in LiOH and NaOH electrolytes.

## 1. Introduction

Because of their distinctive electrochemical performance compared to other energy storage systems, lithium ion batteries (LIBs) with organic electrolyte have been the most successful energy storage device for portable electronics in the last decades and considered as the prime choice to supply power for future electric vehicles, *etc.* [1]. But organic LIBs have several disadvantages: the flammable electrolytes might cause smoke or even fire by inapt using, and they are relatively expensive due to the high-cost electrolytes and special encapsulation. While the emerging of diverse renewable but intermittent energy sources such as solar, tide and wind require cheaper, safer and more sustainable energy storing devices, more and more researchers pay their attention to aqueous battery systems in recent years [2], such as aqueous LIB, aqueous sodium-ion battery(NIB) [3,4,5], aqueous zinc-ion battery [6,7,8] and aqueous aluminum-ion battery [9]. Among those battery systems, aqueous rechargeable alkali-metal ion batteries (ARABs) are more promising not only because they have addressed the hazard and expensive issues, but more importantly, ARABs, as a derivative battery system of LIB, will be developed more rapidly on the basis of the well-developed LIB chemistry.

Due to the limitation of hydrogen and oxygen evolution, the stable window of the aqueous electrolytes is ~1.23 V and much narrower than that of organic electrolytes (3.7 V) [10]. Besides achieving the critical electrochemical performance of specific capacity, cycling and rate capability, the electrode materials for ARABs have to work in a narrow and appropriate potential range to gain a relatively high battery voltage. Numerous materials were investigated as to identify promising anode and cathode electrodes for ARABs. Owing to the intrinsic chemistry characteristics between ARABs and LIB/NIB [11,12], the conventional organic LIB/NIB cathode materials especially the lithium/sodium layered transition metal oxide and its analogs (LiCoO_2_ [13], LiMn_2_O_4_ [14], LiNi_1/3_Co_1/3_Mn_1/3_O_2_ [15], LiNiPO_4_ [16], Na_0.44_MnO_2_ [17], and Na_4_Mn_9_O_18_ [7]) satisfy the comprehensive requirements of ARABs, thus providing a huge cathode reserve for ARABs. Although numerous cathode materials could be considered, the choice for anode materials is still far from optimistic. The widely used Li_4_Ti_5_O_12_ [18] and graphite materials are chemically instable during Li intercalation process in aqueous electrolytes caused by serious water electrolysis. The alloying anode materials for LIB and NIB such as Si, Sb, Sn and their compounds have very high specific capacity, but due to the very low redox potential, they are unqualified for the anode of ARABs. So far, only vanadium based compounds (e.g., VO_2_ (B) [19], LiV_3_O_8_ [20,21], NaV_6_O_15_ [22]), polyanionic compounds (NaTi_2_(PO_4_)_3_ [8,23], TiP_2_O_7_ [24,25], LiTi_2_(PO_4_)_3_ [24]) and some organic compounds (e.g., polyimide [26,27]) were demonstrated as favorable candidates for anode. In a primary research period for electrodes of ARABs, high specific capacity and fitting redox potential would be more important than cycling and rate performance, because actually, we could improve the latter characteristics by morphology, doping, coating and optimization [28,29]. For instance, Li *et al.* proposed a “carbon shell-protection” solution and fabricated binder-free Fe_3_O_4_-C nanorod array anode achieving high capacity (7776 C·cm^−3^), stable cycling performance (>5000 times) and excellent rate capability within a working potential window of −1.3~−0.5 V (*vs.* Ag/AgCl) [30]. With such morphology modification, Fe_3_O_4_ become a very impressive anode material for aqueous battery. Unfortunately, while many cathode materials could work at high potentials, for a large part of the anode materials, their working potentials are not low enough. Therefore, it is urgent to develop new anode materials that have relatively high specific capacity and proper electrochemical potential window.

Bismuth is a semimetal with colorful optical characteristic. As early as 2001, Crosnier *et al.* reported that Bi could be used as anode for organic lithium ion battery [31], but since then, in a long time, Bi has been out of horizon in the field of batteries’ research. Until quite recently, Bi was studied as an alloying anode material for organic Mg-ion battery with theoretical capacity of 385 mAh·g^−1^ [32,33]. And Su *et al.* found that Bi could be utilized as anode for organic Na-ion battery based on the intercalation mechanism via density function theory calculations and could deliver reversible specific capacity of 561 mAh·g^−1^ [34]. Until now, Bi has not been investigated as electrode material for aqueous batteries. In this paper, the energy storage property of Bi electrode in LiOH, NaOH and KOH aqueous solutions is firstly investigated, and we demonstrate that Bi is a promising anode for ARABs with high capacity of 170 mAh g^−1^ and relatively low redox potential range (−1~0 V).

## 2. Results and Discussion

**Figure 1:**
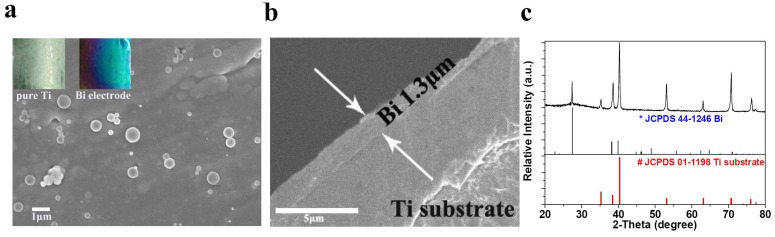
(**a**) Field emission scanning electron microscopy image of bismuth electrode. The insets are the optical pictures of bare substrate and bismuth electrode; (**b**) The cross-sectional scanning electron microscopy (SEM) image and (**c**) X-ray diffraction pattern of bismuth electrode.

The bismuth electrode was synthesized via a simple hydrothermal method, and Bi film was directly grown on a Ti substrate with different colors of purple to blue. The optical difference between Bi electrode and clean Ti substrate without electrode materials is very distinguish, as shown in the inset of Figure 1a. About 1.2–1.3 mg bismuth was loaded on each 2 cm × 2 cm Ti substrate. Bi is homogenously distributed as a thin film at Ti substrate with some carbon spheres on the surface (Figure 1a), and Bi film is mechanically robust upon bending of the substrate. The thickness of Bi film is ~1.3 μm, as shown in Figure 1b. The XRD pattern of Bi electrode is shown in Figure 1c. In addition to the peaks from Ti substrate (JCPDS: 01-1198), only sharp diffraction peak of Bi (JCPDS: 44-1246) can be detected, indicating that impurities such as Bi_2_O_3_ or other bismuth oxides do not exist and the as-prepared Bi film has good crystallinity.

**Figure 2:**
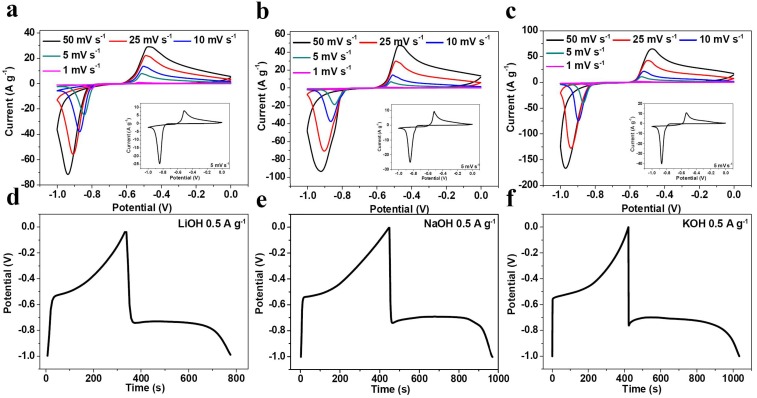
Cyclic voltammograms (CV) curves at different scan rates and in different electrolytes: (**a**) 1 M LiOH; (**b**) 1 M NaOH; (**c**) 1 M KOH. Galvanostatic charge-discharge curves at 0.5 A·g^−1^ in (**d**) 1 M LiOH; (**e**) 1 M NaOH and (**f**) 1M KOH electrolytes.

Figure 2a–c shows cyclic voltammograms (CV) of the synthesized Bi electrode at 1 M LiOH, NaOH and KOH electrolytes at different scan rates within potential range of −1~0 V. From 1 to 50 mV·s^−1^, Bi electrode displays a pair of sharp cathodic and anodic peaks, revealing that Bi has reversible electrochemical feasibility in LiOH, NaOH and KOH alkaline electrolytes. At specific scan rate of 5 mV·s^−1^, as the insets of Figure 2a–c shows, the CV curves are similar in all three electrolytes with reduction and oxidation potentials at around −0.85 and −0.52 V (*vs.* SCE), respectively, which are in good agreement with the electrochemical characteristics of Bi_2_O_3_ electrodes in aqueous alkaline electrolytes [35]. The similarity suggests that the energy storage mechanism of Bi and Bi_2_O_3_ in aqueous electrolytes would be similar [36]. That is, the cathodic process and anodic process correspond to the reduction of Bi^3+^ and oxidation of Bi, respectively. In aqueous electrolytes, when Bi reacts completely, with 6 electrons participating in the Equation (1), the theoretical capacity would be 384.7 mAh·g^−1^.

Bi(s) → Bi^3+^(s) + 3e^−^ (1)

As Figure 2d–f shows, at the current density of 0.5 A·g^−1^, although the galvanostatic charge-discharge curves are similar in three different electrolytes, the Bi electrode in LiOH, NaOH and KOH electrolytes delivers discharge specific capacity of 121, 145 and 170 mAh·g^−1^ within potential range of −1~0 V, respectively. The capacity value in KOH electrolyte is comparable to the theoretical capacity of well-known Li_4_Ti_5_O_12_ anode (175 mAh·g^−1^). With high capacity and low redox potential, Bi is suitable to be used as promising anode material for ARABs.

**Figure 3:**
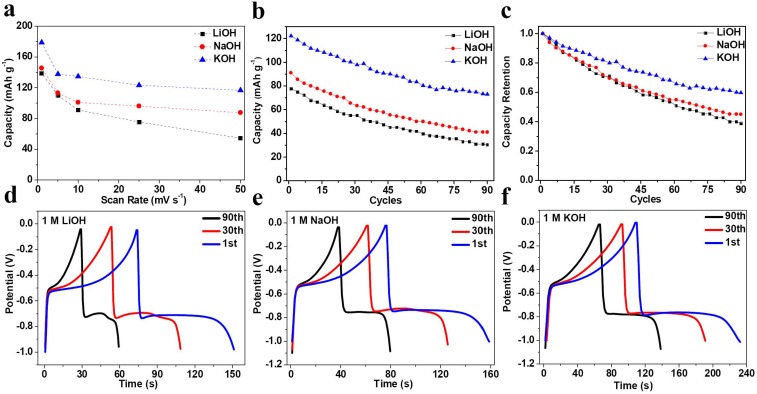
(**a**) Rate performance of Bi electrode at various scan rates; (**b**) Cycling performance and (**c**) Capacity retention of Bi electrode in 1 M LiOH, NaOH and KOH electrolytes at 2 A·g^−1^. Galvanostatic charge-discharge curves of the first, 30th and 90th cycle in (**d**) 1 M LiOH; (**e**) 1 M NaOH and (**f**) 1 M KOH electrolytes.

The CV rate capability in three alkaline electrolytes were calculated and shown in Figure 3a. At 1 mV·s^−1^, the specific capacity in 1 M LiOH, NaOH and KOH electrolytes are 139, 146 and 179 mAh·g^−1^, respectively, and at 50 m·V·s^−1^, the capacity in LiOH electrolyte quickly drops to 54 mAh·g^−1^, and is much smaller than 87 and 117 mAh·g^−1^ in NaOH and KOH electrolytes, respectively. With scan rate increased from 1 to 50 m·V·s^−1^, the capacity retention in LiOH, NaOH and KOH electrolytes are 38.8%, 59.5% and 65.4%, respectively. This indicates that in CV testing, specific capacity and rate capability in KOH electrolyte are the best, and those in NaOH solution are better than in LiOH. Taking into account the specific capacity of Bi electrode at 0.5 A·g^−1^ in three alkaline electrolytes, it seems that the electrochemical characteristics have strong relationship with the preference of these solutions. To confirm this hypothesis, galvanostatic charge-discharge cycling was applied to Bi electrode in three kinds of electrolytes, as shown in Figure 3b,c. Obviously, at the current density of 2 A·g^−1^, cycling performance in KOH electrolyte is much better than that in other two electrolytes, and the capacity in NaOH electrolyte is ~10 mAh g^−1^ higher than that in LiOH solution at each specific cycle. We recorded the first, 30th and 90th cycles in three electrolytes, as displayed in Figure 3d–f. In the first cycle, Bi electrode in LiOH, NaOH and KOH electrolytes delivers specific capacity of 78, 91 and 122 mAh·g^−1^, respectively. In the 30th cycle, the capacity could be 55, 63 and 98 mAh·g^−1^, respectively. While in 90th cycle, the capacity remains 30, 41 and 73 mAh·g^−1^ with capacity retention of 38.5%, 45% and 60%, respectively. All above results suggest that the electrochemical properties such as specific capacity, rate capability and cycling stability in KOH electrolyte is much better than that in both NaOH and LiOH electrolytes, and LiOH electrolyte is the worst among the three tested electrolytes.

To testify the mobility of the alkali metal ions, we carried out the electrochemical impedance spectroscopy (EIS) to investigate the kinetics of Bi electrode in ARABs. Alkali-ion diffusion coefficient is one of the most important kinetic parameters that govern electrochemical characteristics of ARABs. Ion diffusion coefficient was calculated according to the straight line at low frequencies of Nyquist plots based on Equations (2) and (3):

(2) $$D=\frac{R^{2}T^{2}}{2A^{2}n^{4}F^{4}C^{4}\sigma^{2}}$$

*Z*_Re_* = R*_s_* + R*_ct_*+* σω*^−^*^1/2^ (3)

**Figure 4:**
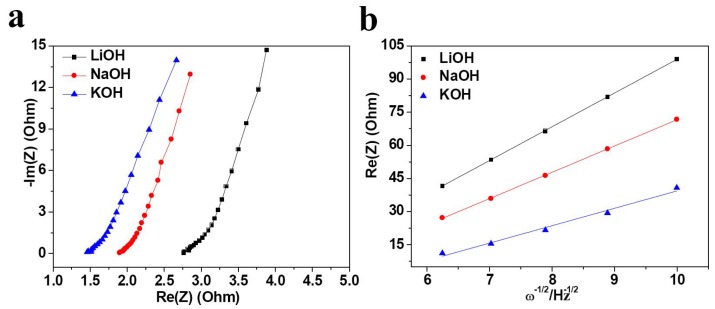
(**a**) Impedance spectra of Bi electrode and (**b**) slope of the plots of *Z*_Re_ against ω^−1/2^ in 1 M LiOH, NaOH and KOH electrolytes.

In this two equations, *R*, *T*, *A*, *n*, *F*, *C* and σ correspond to gas constant, room temperature, surface area of the electrode, the electron numbers that ion transferred, Faraday constant, the ion’s concentration and the slope *Z_Re_* against ω*^−^*^1/2^, respectively. The plots *Z_Re_* against ω*^−^*^1/2^ is shown in Figure 4b.

The Nyquist plots were extracted from EIS data; the ohimic resistance (*R*_s_) represents total resistance of Bi thin film, electrolyte and the contact between electrolyte and Bi electrode. As shown in Figure 4a and Table 1, *R*_s_ with LiOH, NaOH and KOH electrolytes is 2.76, 1.89 and 1.45 Ω, respectively. The ion diffusion coefficients of three alkali hydroxide electrolytes are presented in Table 1 and increased in the following order: LiOH < NaOH < KOH. For the pseudocapacitive systems, in order to keep quick redox process, facile electron transport and fast diffusion of ions in the electrode is needed, as demonstrated by Fic *et al.* [37]. Because in KOH electrolyte the *R*_s_ is the smallest while ion diffusion is the fastest, it is reasonable that electrochemical properties such as specific capacity, cycling stability and rate capability of Bi film electrode in KOH electrolyte are the highest or the best, and performance in NaOH electrolyte is better than that in LiOH electrolyte.

**Table 1:** Impedance parameters evaluated from electrochemical impedance spectroscopy (EIS).

Electrolyte	*R*_s_ (Ω)	σ	*D* (×10^−1^^1^ cm^2^·s^−1^)
LiOH	2.76	15.28	1.67
NaOH	1.89	11.90	2.78
KOH	1.45	7.87	6.35

## 3. Experimental Section

### 3.1. Sample Preparation and Characterization

A facial hydrothermal route was employed by using bismuth nitrate pentahydrate (AR, ≥99%), ethylene glycol (AR, ≥99%), glucose (AR) and acetone (AR, ≥99.5%) to fabricate binder-free bismuth thin-film electrode. In a typical synthesis, 0.6 g bismuth nitrate pentahydrate and 2 g glucose was dispersed in 12 mL acetone and 6 mL ethylene glycol by magnetic stirrer for 30 min. The transparent dispersed solution with a 2 cm × 2 cm Ti substrate was transferred into a Teflon-lined autoclave and maintained at 180 °C for 8 h. After the reaction, the Ti substrate was collected and washed with deionized water; the bismuth electrode was finally obtained by drying the substrate for 12 h at 60 °C. The electrode was characterized by field emission scanning electron microscopy (SEM, JSM-6700F, 5 kV, Electron Company, Tokyo, Japan) and X-ray diffraction (XRD, D-8 Avance, Bruker Corporation, Ettlingen, Germany).

### 3.2. Electrochemical Characterization

All electrochemical testing was performed by a standard three-electrode cell setup with bismuth electrode, platinum plate and saturated calomel electrode as working, counter and reference electrode on CHI 760 D electrochemical workstation at room temperature. The actual testing area was 3 cm^2^, and all potentials mentioned in this article were *vs.* SCE. Testing electrolytes were 1 M LiOH, NaOH and KOH. EIS measurements were performed by applying an AC voltage with 5 mV amplitude in a frequency range from 0.05 Hz to 100 kHz. The specific capacity *C* (mAh) from cyclic voltammograms was calculated according to Equation (4):

(4) $$C=\frac{\int I\times dE}{3.6\times \upsilon \times m}$$

where *I* (A) and *dE* (V) are current, differential potential and υ (V·s^−1^), *m* (g) are the sweep rate, mass of Bi film, respectively. And specific capacity from galvanostatic charge-discharge testing was calculated according to Equation (5):

(5) $$C=\frac{I\times \Delta t}{m}$$

where *I* (mA), Δ*t* (h) and *m* (g) represent the current, discharging time and mass of Bi film, respectively.

## 4. Conclusions

We firstly demonstrated that bismuth is a promising anode material for ARABs with relatively low redox potential (reduction and oxidation potentials at −0.85 and −0.52 V *vs.* SCE) and high capacity (170 mAh·g^−1^ in KOH electrolyte). The electrochemical performance such as capacity, cycling stability and rate capability in alkali metal hydroxide electrolytes increased according to the following order: LiOH < NaOH < KOH. By EIS testing, we found that the *R*_s_ and ion diffusion of Bi electrode in KOH solution are the best, while the performance in LiOH electrolyte is worse than that in NaOH electrolyte. It was confirmed that faster electron and ion transfer/diffusion ensure quicker redox process and provide better electrochemical properties. Our work not only develops a new prospective anode for safe and inexpensive battery system but also provides insights on the electrolyte influence on the performance of ARABs.
